# Assessing mental fatigue in football: a systematic review

**DOI:** 10.3389/fspor.2026.1861469

**Published:** 2026-06-09

**Authors:** Diogo Aveiro, Francisco Campos, Rita Neves Rodrigues, Fernando Martins, John Kiely

**Affiliations:** 1 Department of Physical Education & Sport Sciences, University of Limerick, Limerick, Ireland; 2 Polytechnic University of Coimbra, Coimbra, Portugal; 3 SPRINT - Sport Physical Activity and Health Research & INnovation CenTer, Polytechnic University of Coimbra, Coimbra, Portugal; 4 inED - Centre for Research & Innovation in Education, Polytechnic University of Coimbra, Coimbra, Portugal

**Keywords:** cognitive performance, decision-making, football performance, mental fatigue, physiological monitoring

## Abstract

**Background:**

Mental fatigue is a psychophysiological state that can impair cognitive, technical, tactical, and physical performance in football. Despite growing interest in this topic, there is currently no standardized approach for assessing and monitoring mental fatigue in football contexts. This systematic review aimed to (i) synthesize the evidence regarding the effects of mental fatigue on football performance and (ii) identify the methodologies currently used to induce, detect, and analyze mental fatigue in football.

**Methods:**

Following PRISMA 2020 guidelines, a systematic search was conducted in the PubMed, Web of Science, SportDiscus, Scopus, and Springer Nature Link. Studies published between 1 January 2014 and 15 May 2026 in English, Portuguese, Spanish or French were included. Methodological quality was assessed using the Mixed Methods Appraisal Tool. Twenty-eight studies met the eligibility criteria.

**Results:**

The findings showed that mental fatigue generally impairs football performance, particularly decision-making, technical execution, tactical behavior, attention, and perceived exertion. However, the magnitude and consistency of these effects varied according to the type of performance outcome assessed and the methodological approaches used across studies. Subjective assessment tools, such as visual analogue scales and perceived workload measures, were the most commonly used methods, whereas objective approaches, including eye-tracking systems, functional near-infrared spectroscopy, and psychomotor vigilance tests, were less frequently applied. Overall, substantial methodological heterogeneity and the absence of standardized assessment protocols limited comparisons between studies and reduced the practical app licability of findings.

**Conclusion:**

Current evidence suggests that mental fatigue negatively affects multiple dimensions of football performance, although methodological inconsistencies remain substantial. There is a clear need for standardized, ecologically valid assessment and monitoring protocols integrating both subjective and objective measures to improve performance management and recovery strategies in football.

## 1 Introduction

Mental fatigue (MF) is a psychophysiological state resulting from prolonged periods of demanding cognitive activity, typically characterized by feelings of tiredness, reduced cognitive efficiency, impaired attentional regulation, and increased perceived effort (1). In the present review, MF was operationally defined as a state induced directly through cognitive-demanding tasks or indirectly inferred through conditions associated with sustained cognitive strain, impaired recovery, or elevated mental workload. During mentally fatiguing events, the elevated attentional and decision-making demands associated with cognitive effort alter the underlying neurophysiological environment, increasing perceived effort, reducing motivation, and impairing movement regulation (Boksem and Tops, 2008; Marcora et al., 2009 (1);. Collectively, these psychophysiological alterations may negatively influence football players’ on-field performance through increased perceived effort, reduced motivation, and impaired movement regulation (2). Consequently, prior evidence illustrates that MF hinders critical dimensions of psychomotor performance, such as passing accuracy and decision-making (3). Smith et al. (2016), for example, reported that MF decreases passing accuracy and speed of shots, while also impairing inter-player synchronization during defensive actions. Subsequently, in football, MF appears to exert a direct impact on both individual performances and the collective performance of the team (4). However, the evidence regarding the effects of MF on football performance remains methodologically heterogeneous and insufficiently synthesized (1).

While research investigating the potential impact of MF on sports performance has increased substantially in recent years (1, 3, 5), more recent systematic reviews and meta-analytical evidence continue to highlight the negative effects of MF on sport-specific performance and the methodological challenges associated with its induction and assessment (6–8). The absence of standardized MF assessment approaches inhibits the capacity of coaches and performance support staff to productively design monitoring processes capable of guiding training and/or recovery interventions. The need for a deeper understanding of MF has been noted by Kunrath (5), who proposed that several different methodologies should be deployed to assess MF. Similarly, Van Cutsem et al. (1) underlined the need to assess the cognitive effort of athletes, and suggested a combination of physiological measures (e.g., pupillary behavior) and behavioral measures (e.g., performance in targeted football tasks) to enhance the specificity and accuracy of MF monitoring. Against this backdrop, this systematic literature review has two main objectives:

- To synthesize the evidence regarding the effects of MF on football performance across physical, technical, tactical, and cognitive domains.
- To analyze the methodologies currently used to induce, assess, and monitor MF in football contexts.

By addressing these objectives, this review aims to bridge the critical gap between emerging scientific insights and practical application; thereby providing practitioners with an evidence-based framework for detecting, monitoring, and managing MF in competitive environments. Recent protocol-based work has also started to address this gap by proposing football-specific, multimodal designs that combine subjective neurophysiological, visual, and performance-based measures within an ecologically grounded framework (9).

## 2 Methods

### 2.1 Search strategy

This systematic review was conducted according to PRISMA 2020 guidelines (10). The review was not prospectively registered, and no formal review protocol was prepared. To conduct this systematic review, scientific articles were collected in the first week of November 2024 using PubMed, Elsevier's Scopus, Clarivate's Web of Science, SportDiscus and Springer Nature Link data-bases. An updated search was subsequently conducted on 9 December 2024 across all databases to ensure inclusion of the most recent eligible studies. A further updated search was conducted on 15 May 2026 across all databases to identify additional eligible studies published between 10 December 2024 and 15 May 2026. The same core Boolean search equation was applied across all databases: “mental fatigue” AND (“football” OR “soccer”) AND “performance” AND (“monitor*” OR “assess*” OR “track*”). To ensure conceptual consistency while accounting for database-specific search interfaces, equivalent Boolean logic was maintained across databases whenever supported by the platform interface. The search interfaces and field restrictions were adapted to the closest available searchable fields in each platform. PubMed was searched using the general search field with automatic term mapping; Scopus was searched in Article title, Abstract, and Keywords; Web of Science was searched using Topic; SportDiscus via EBSCOhost was searched using the general search field; and Springer Nature Link was searched using its general search interface. Springer Nature Link does not provide an equivalent searchable Title/Abstract/Keyword field combination within the interface used, the general search function was applied only for this database. To reduce the risk of retrieving conceptually irrelevant full-text matches, database-level filters were applied for content type, discipline, subdiscipline, language, and publication date, and all retrieved records were subsequently screened using the same eligibility criteria as the other databases. Because field-tag options and indexing structures differ across databases, the exact search fields, field tags, filters, date limits, search dates, updated periods, and records retrieved for each database are reported in Supplementary Table S1. This approach was adopted to maximize transparency and reproducibility while maintaining conceptual consistency across databases. The search strategy prioritized the explicit term “mental fatigue” to maximize conceptual specificity and reduce the inclusion of studies addressing broader cognitive or psychological constructs not directly aligned with the operational framework adopted in this review. However, related constructs such as cognitive load, mental strain, cognitive effort, and recovery-related cognitive fatigue were considered during eligibility screening and full-text assessment when conceptually relevant to the review objectives.

For Springer Nature Link, the following filters were applied according to the options available in the database interface: Content Type – Article; Discipline – Medicine & Public Health; Subdiscipline – Orthopedics, Rehabilitation Medicine, Human Physiology, Metabolic Diseases; Language – English, the other language options did not meet our criteria. The search included articles published between 1 January 2014 and 15 May 2026 in English, Portuguese, Spanish, and French to capture recent and methodologically up-to-date publications. The original 10-year publication window was adopted to prioritize contemporary evidence reflecting recent methodological developments in MF induction, monitoring technologies, and football-specific performance analysis, and was subsequently extended through the final update search to maintain the review's timeliness. The complete database-specific search strategies, including search strings, field tags, filters, language restrictions, search dates, data limits, updated periods, and records retrieved for each database, are provided in Supplementary Table S1.

### 2.2 Eligibility criteria

An eligibility-criteria was devised to capture studies that directly contribute to research related to monitoring MF in football. The studies were published between 1 January 2014 and 15 May 2026; they investigated MF, cognitive effort, and cognitive functioning related to football. Studies were eligible if they directly assessed or experimentally induced MF, or if they investigated closely related constructs (e.g., cognitive load, cognitive effort, mental strain, or recovery-related cognitive fatigue) considered relevant to the operational framework of MF adopted in this review. They were published in English, Portuguese, Spanish, or French. Eligible studies also included qualitative and quantitative investigations describing methods for monitoring, identifying, or analyzing MF in competitive or training contexts involving football players. During eligibility screening, related terms such as ‘mental load,’ ‘cognitive performance,’ ‘mental exertion,’ ‘cognitive effort,’ ‘mental strain,’ ‘cognitive fatigue’ and other synonyms were considered when they were conceptually aligned with the operational definition of MF adopted in this review. Conversely, the exclusion criteria consisted of studies that were published prior to the year 2014, studies with no available full text, academic theses, non-scientific books or articles and studies that were solely focused on physical fatigue with no relation to MF. Studies that did not describe a method of identifying and analyzing MF and those focusing on sports other than football or other participants, such as coaches or referees, were also excluded.

**Figure 1:**
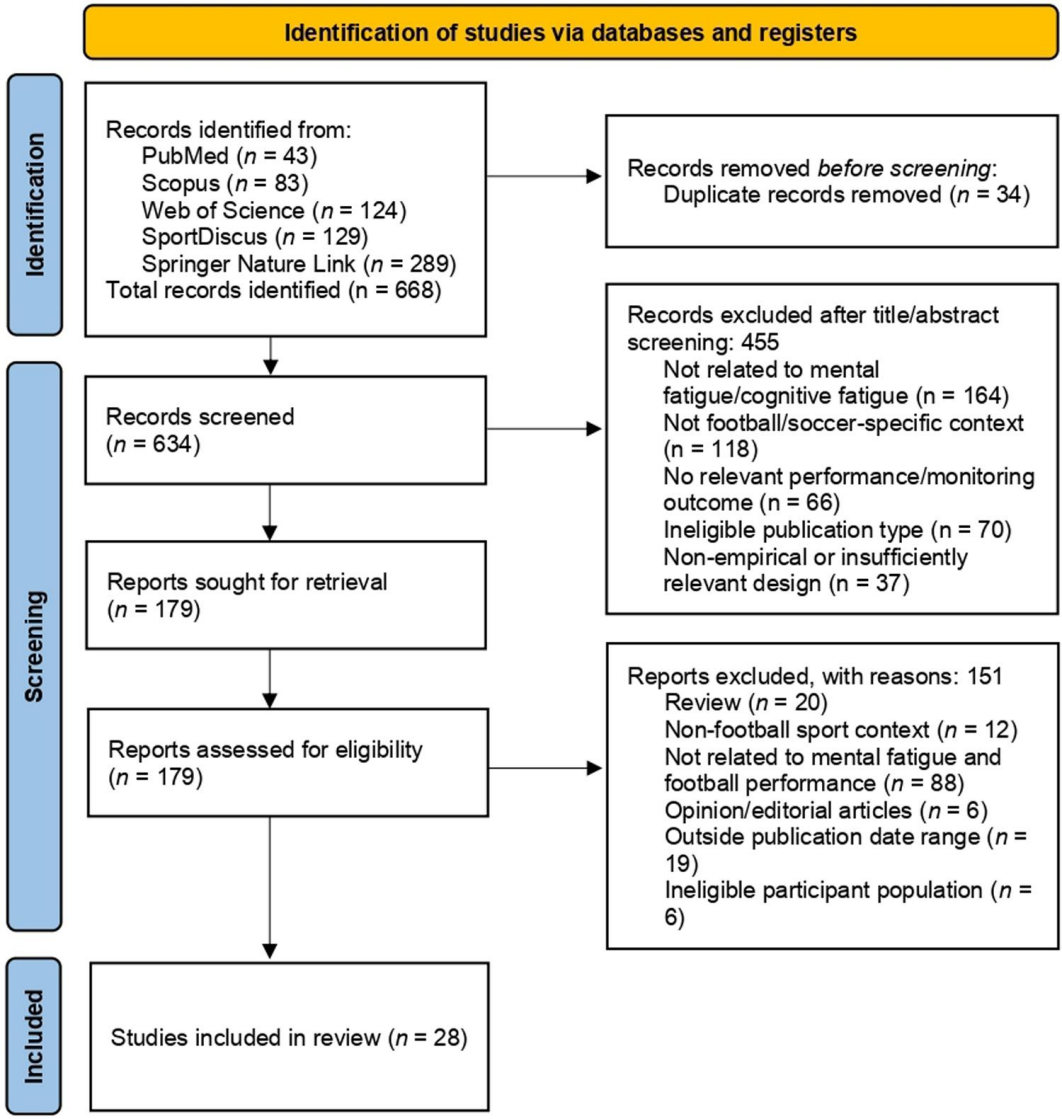
PRISMA flowchart (10).

The study selection process was conducted in sequential stages. Using the predefined search equation, records were identified across PubMed, Scopus, Web of Science, SportDiscus, and Springer Nature Link. Duplicate records were removed using reference management tools. Titles and abstracts were then screened independently according to the predefined eligibility criteria. Records excluded at this stage were categorized according to the primary reason exclusion. Full-text reports were subsequently retrieved and assessed for eligibility, and reasons for exclusion were recorded. The complete study selection process, including the number of records identified, screened, excluded, assessed for eligibility, and included in the final review, is presented in Figure 1 and summarized in the Results section.

### 2.3 Quality assessment

The methodological quality of the included studies was assessed using the Mixed Methods Appraisal Tool (MMAT), version 2018 (11). The MMAT was selected because the included studies presented methodological heterogeneity, including randomized, non-randomized, crossover, quasi-experimental, and descriptive quantitative designs. Therefore, a flexible appraisal tool applicable across multiple methodological frameworks was considered more appropriate than risk-of-bias instruments developed exclusively for randomized controlled trials.

To facilitate descriptive comparison across studies with different methodological designs, MMAT criteria were converted into numerical values (1 = criterion met; 0.5 = unclear or partially met; 0 = criterion not met). This scoring approach was used exclusively for descriptive purposes and not as a formal quantitative validation procedure, in line with the MMAT guidance discouraging the calculation of overall quality scores. The numerical conversion of MMAT criteria was used only for descriptive comparison and did not inform study weighting, exclusion decisions, or quantitative synthesis. Based on the cumulative descriptive scores, studies were categorized as low, medium, or high methodological quality to facilitate interpretation of the evidence. The results of the methodological quality assessment are presented in Table 1.

**Table 1:** Evaluation of articles according to the mixed methods appraisal tool (MMAT).

Studies	Screening questions		1. Qualitative					2. Quantitative randomized controlled trials					3. Quantitative nonrandomized					4. Quantitative descriptive					5. Mixed methods					
	S1.	S2.	1.1.	1.2.	1.3.	1.4.	1.5.	2.1.	2.2.	2.3.	2.4.	2.5.	3.1.	3.2.	3.3.	3.4.	3.5.	4.1.	4.2.	4.3.	4.4.	4.5.	5.1.	5.2.	5.3.	5.4.	5.5.	Score
Alder et al. (39)	Y	Y											Y	Y	Y	CT	Y											M
Angius et al. (17)	Y	Y						Y	Y	Y	CT	Y																H
Badin et al. (18)	Y	Y						Y	Y	Y	CT	Y																H
Brett et al. (27)	Y	Y																Y	CT	Y	CT	Y						M
Coutinho et al. (20)	Y	Y											Y	Y	Y	CT	Y											H
Coutinho et al. (21)	Y	Y											Y	Y	Y	CT	Y											H
Da Silva et al. (35)	Y	Y											Y	Y	CT	N	Y											M
Da Silva et al. (36)	Y	Y											Y	Y	Y	CT	Y											H
Donnan et al. (22)	Y	Y						CT	Y	Y	CT	Y																M
Filipas et al. (15)	Y	Y											Y	Y	Y	CT	Y											H
Fortes et al. (30)	Y	Y						Y	Y	Y	CT	Y																H
Fortes et al. (31)	Y	Y						Y	Y	Y	CT	Y																M
Fortes et al. (41)	Y	Y						Y	Y	Y	CT	Y																H
Gantois et al. (32)	Y	Y						Y	Y	Y	CT	Y																H
Greco et al. (16)	Y	Y						Y	Y	Y	CT	Y																H
Kunrath et al. (37)	Y	Y											Y	Y	Y	CT	Y											M
Nishida et al. (33)	Y	Y						Y	Y	Y	CT	Y																H
Rubio-Morales et al. (42)	Y	Y						Y	Y	CT	CT	Y																M
Silva et al. (38)	Y	Y						Y	Y	CT	CT	Y																M
Skoki et al. (23)	Y	Y											Y	Y	Y	CT	Y											H
Smith et al. (29)	Y	Y						Y	Y	Y	CT	Y																H
Smith et al. (40)	Y	Y						Y	Y	Y	CT	Y																H
Smith et al. (19)	Y	Y						Y	Y	Y	CT	Y																H
Soltani et al. (34)	Y	Y						Y	Y	Y	Y	Y																H
Soylu and Arslan (24)	Y	Y						Y	Y	Y	N	Y																M
Soylu et al. (25)	Y	Y											Y	Y	Y	CT	Y											M
Soylu et al. (26)	Y	Y						Y	Y	Y	Y	Y																H
Staiano et al. (28)	Y	Y						Y	Y	Y	CT	Y																H

Y, Yes; N, No; CT, Cannot tell. Y-1, CT-0.5, N-0. Score: Low-0-2, Medium-3-4. High-5-7. L, low methodological quality; M, medium methodological quality; H, high methodological quality. Items: S1. Are there clear research questions? 2. Do the collected data allow to address the research questions? 1.1. Is the qualitative approach appropriate to answer the research question? 1.2. Are the qualitative data collection methods adequate to address the research question? 1.3. Are the findings adequately derived from the data? 1.4. Is the interpretation of results sufficiently substantiated by data? 1.5. Is there coherence between qualitative data sources, collection, analysis and interpretation? 2.1. Is randomization appropriately performed? 2.2. Are the groups comparable at baseline? 2.3. Are there complete outcome data? 2.4. Are outcome assessors blinded to the intervention provided? 2.5. Did the participants adhere to the assigned intervention? 3.1. Are the participants representative of the target population? 3.2. Are measurements appropriate regarding both the outcome and intervention (or exposure)? 3.3. Are there complete outcome data? 3.4. Are the confounders accounted for in the design and analysis? 3.5. During the study period, is the intervention administered (or exposure occurred) as intended? 4.1. Is the sampling strategy relevant to address the research question? 4.2. Is the sample representative of the target population? 4.3. Are the measurements appropriate? 4.4. Is the risk of nonresponse bias low? 4.5. Is the statistical analysis appropriate to answer the research question? 5.1. Is there an adequate rationale for using a mixed methods design to address the research question? 5.2. Are the different components of the study effectively integrated to answer the research question? 5.3. Are the outputs of the integration of qualitative and quantitative components adequately interpreted? 5.4. Are divergences and consistencies between quantitative and qualitative results adequately addressed? 5.5. Do the different components of the study adhere to the quality criteria of each tradition of the methods involved? MMAT scores were used for descriptive comparison only and did not inform study weighting, exclusion decisions, or quantitative synthesis.

### 2.4 Reviewing studies and collecting data

 Table 2 summarizes the main characteristics of the 28 articles in this systematic review both methodological information (authors, year, participants, intervention, instruments and variables) and summary of key findings (objectives, main results and conclusions). This systematic review aimed to identify studies reporting specific approaches to identifying and analyzing football-related MF. Nevertheless, it also included studies where MF was not explicitly related to performance, but variables that may lead to MF had been manipulated and thus affected performance. An example of this is studies whose main focus dealt with sleep but whose effects could be related to MF among athletes.

**Table 2:** Structured summary of the included studies.

Reference	Title	Objectives	Participants	Intervention	Instruments or Variables		Main results and conclusions
					MF	Performance	
Alder et al. (39)	The combination of physical and mental load exacerbates the negative effect of each on the capability of skilled soccer players to anticipate action.	To evaluate how the combination of physical and mental load affects the anticipatory decisions of skilled SP.	16 adult male SP. (*M* = 22.4 years; *SD* = 2.5)	SP performed an 11 *vs.* 11 video-based anticipation test under four conditions: baseline, physical load, mental load (30 min ST), and combined load (Stroop during physical task). Response accuracy, visual search behavior, and perceived effort were assessed.	RSME and RPE.	RA, anticipation performance and VS.	Physical and mental loads impair skilled SP anticipatory judgments, with combined loads amplifying this effect. This impairment is attributed to the reduced ability to allocate cognitive resources to relevant task information.
Angius et al. (17)	Physical and MF reduce psychomotor vigilance in professional football players.	To examine how MF affects RSA and to assess the impact of both physical fatigue and MF on psychomotor vigilance.	17 male professional SP (Goalkeepers were excluded). (*M* = 26 years; *SD* = 2)	SP followed 30 min of ST (MF condition) or control with RSA tests. Speed while running, HR, brain oxygenation, and perceived exertion were monitored. A PVT measured attention and reaction times before and after tasks.	RPE, NASA-TLX, HR, NIRS, AND PVT.	RSA.	MF does not impair RSA among professional SP. However, both physical and MF negatively impact psychomotor vigilance, suggesting that strategies to mitigate both forms of fatigue should be implemented to enhance performance in SP.
Badin et al. (18)	MF: Impairment of technical performance in small-sided soccer games.	To investigate how MF impacts both physical abilities and technical skills during soccer SSG.	20 young male SP from an Australian National Premier League club. (*M* = 17.8 years; *SD* = 1)	SP completed 30 min of a computerized ST (MF) or watched a neutral documentary (control), then played 15 min 5v5 SSG. Technical and physical performance, HR, perceived exertion, and subjective ratings of fatigue, effort, and motivation were assessed.	VAS.	Technical performance during SSG, physical activity profile, HR and RPE.	MF increased subjective fatigue and perceived effort and impaired selected technical actions during SSG, whereas most physical-performance variables were minimally or inconsistently affected.
Brett et al. (27)	Analysis of mental and physical fatigue over the course of a professional English Premier League season in outfield players	To examine how MF and PF varied at group and individual levels across a full English Premier League season.	21 male first-team EPL outfield players across a 40-week season, 151 training sessions and 46 matches. (M = 25.3 years; SD = 3.8)	No experimental MF induction. Prospective cohort monitoring study. Players rated MF and PF on match day minus one across 46 timepoints. The season was divided into early, middle and late phases.	100-mm VAS for subjective MF; 100-mm VAS for PF.	No direct performance test; fatigue was monitored in relation to season phase and match/training exposure.	No significant group-level changes in MF or PF were observed across the season; however, substantial inter-individual variability was identified, supporting individualized monitoring of fatigue-related responses in applied football contexts.
Coutinho et al. (20)	MF and spatial references impair SP’ physical and tactical performances.	To assess MF and the presence of additional corridor and sector lines on the pitch influenced players’ physical and tactical performance during SSG.	12 highly trained amateur youth male SP. (*M* = 15.9 years; *SD* = 0.8)	SP took part in four 6v6 SSG. One team completed a motor coordination task that induced MF; the other team a control task. Games were played with or without pitch reference lines. Physical (GPS data, accelerations, CMJ) and tactical performance (positional synchronization, spatial metrics) were assessed.	VAS.	GPS variables, accelerations, CMJ and tactical synchronization metrics.	MF harms players’ physical and tactical performance in SSG, reducing environmental awareness and positioning. Reference lines can also confuse decision-making. This suggests that coaches may be able to use the temporal ordering of MF and having a variable scope of pitch design to promote adaptability in youth SP.
Coutinho et al. (21)	Exploring the effects of mental and muscular fatigue in SP’ performance.	To investigate how mental and added muscular fatigue impact the physical performance and tactical behavior of soccer players.	10 amateur youth male SP. (*M* = 14.3 years; *SD* = 0.6)	SP implemented controlled 5 *vs.* 5 SSG under conditions of muscular fatigue (change-of-direction task) and MF (30-minute ST). Time-motion and tactical behavior were analyzed using positional data.	RPE and VAS.	Time-motion variables, positional data and tactical synchronization.	MF hinders athletes’ physical performance and team coordination, indicating the importance of controlling cognitive load before matches or team-dynamic-focused training.
Da Silva et al. (35)	Influence of pre-induced MF on tactical behavior and performance among young elite sp.	To examine how pre-induced MF impacts the tactical behavior and performance of elite male Brazilian SP aged under 17 years.	18 under-17 male Brazilian elite SP. (*M* = 17.1 years; *SD* = 0.5)	SP performed 30 rounds of a ST to induce fatigue before completing a 3 *vs.* 3 tactical assessments using the FUT-SAT. Performances were compared using statistical tests, and fitness protocols.	VAS.	FUT-SAT tactical behavior and performance variables.	SP performed fewer offensive and defensive actions under MF but increased action efficiency and tactical performance, suggesting selective adaptation.
Da Silva et al. (36)	MF in football: Behavioral responses of players with high and low tactical performance.	To evaluate the tactical actions of players with high and low tactical performance in two different scenarios: (1) experiencing MF and (2) not experiencing MF.	18 under-17 male football players	U17 players were assessed in 3v3 games using the FUT-SAT, with and without prior MF induced by a 30 min incongruent ST. Tactical actions were filmed and analyzed using SSA based on ten tactical principles. Players were grouped by tactical performance to compare behavioral responses under both conditions.	VAS.	FUT-SAT.	SP with higher tactical performance performed more offensive and defensive actions in the non-MF condition than in the MF condition and generally outperformed lower tactical-performance players across conditions.
Donnan et al. (22)	The multifaceted implications of mental fatigue on women's football players’ performance in small-sided games	To examine whether 30-min social media use induces mental fatigue and affects mood, attentional focus, communication, perceived exertion, and physical performance during women's football SSGs.	14 women's National League football players M = 25.9 years; SD = 5.9)	Counterbalanced crossover design with two conditions: 30-min social media use to induce MF vs. 30-min seated control condition without phone access, followed by 3 × 7-min 7v7 SSGs.	VAS-MF; BRUMS; think-aloud; communication analysis	GPS; RPE; SSG locomotor output; performance-related verbalizations.	Social-media-induced MF subjective fatigue and negatively affected mood, performance-related thoughts and external communication, but did not meaningfully impair GPS-derived physical performance or RPE during SSGs.
Filipas et al. (15)	Effects of MF on soccer-specific performance in young players.	To examine how MF influences soccer-specific physical and technical performance in young players.	36 young male SP: 12 U14, 12 U16 and 12 U18. (*M* = 15.4 years; *SD* = 0.5)	SP were assessed for physical performance using the Yo-Yo IR1 and technical capabilities using the LSPT and LSST. MF was induced via a 30-minute ST, and a control group performed a 15-minute neutral task.	VAS and NASA-TLX.	Yo-Yo IR1, LSPT and LSST.	MF reduced Yo-Yo IR1 performance across age groups, with larger impairments in older players; LSPT was impaired mainly in U18, whereas LSST was not significantly affected.
Fortes et al. (30)	Effect of exposure time to smartphone apps on passing decision-making in male soccer athletes.	To analyze the effect of exposure time to smartphone applications on passing decision-making performance in professional SP.	20 male professional SP from a Brazilian Third Division team. (M = 24.7 years; SD = 3.6)	SP completed four randomized within-subject conditions: control, 15-min smartphone use, 30-min smartphone use, and 45-min smartphone use before a simulated soccer match. MF was assessed before and after each condition using the ST.	Smartphone-app exposure and Stroop-based MF assessment.	GPAI-derived Decision-Making Index.	At least 30 min of smartphone-app exposure caused MF and impaired passing decision-making performance in mal soccer athletes, suggesting that coaches should control smartphone exposure time before performance contexts.
Fortes et al. (31)	The effect of smartphones and playing video games on decision-making in SP: A crossover and randomised study.	To analyze how engaging with social media on smartphones or playing video games influences the passing decision-making abilities of professional SP.	25 male professional SP. (*M* = 21.8 years; *SD* = 3.1)	SP completed three conditions: control (watching ads), smartphone (30 min of social media), and video game (30 min of FIFA). MF was measured pre- and post-condition via ST. Players then performed a full 90-min simulated match, and passing decision-making was analyzed using video footage and GPAI tool.	Smartphone/social media use and videogame exposure; RPE; HR-related monitoring; TQR, UCI and BL.	DMI.	Using social networks on smartphones and playing video games right before official soccer matches can impair passing decision-making performance in professional SP.
Fortes et al. (41)	Effect of MF on decision-making skill during simulated congested match schedule in professional soccer athletes.	To analyze how MF is induced by playing videogames right before matches affects the decision-making abilities of professional SP during a SCMS.	16 male professional SP. (*M* = 25.3 years; *SD* = 1.1)	SP completed four consecutive simulated matches across four days. Before each match, they played 60 min of FIFA 19 to induce MF. Decision-making skill (accuracy and response time) was assessed pre- and post-gaming using a film-based soccer-specific task. MF was measured by VAS and ST, and internal match load was recorded after each match.	60-min FIFA 19 videogame protocol; VAS for MF; ST/manipulation checks.	Decision-making accuracy and response time.	Videogame-induced MF impaired decision-making response time across days, especially day 3, but did not significantly affect accuracy.
Gantois et al. (32)	Effects of MF on passing decision-making performance in professional soccer athletes.	To examine how MF influences the decision-making performance related to passing in elite SP.	20 male professional SP. (*M* = 22.6 years; *SD* = 3.3)	In a crossover design, SP completed three conditions (control, 15 min ST, 30 min ST), across three weeks. MF was assessed via ST before and after each condition. Passing decision-making was evaluated using the GPAI throughout a 90 min match.	ST.	GPAI during 90-min match play	A 30-min ST impaired passing decision-making during a 90-min training match compared with 15-min ST and control.
Greco et al. (16)	Negative effects of smartphone use on physical and technical performance of young footballers.	To explore how extended smartphone usage affects the physical and technical abilities of young football players.	16 male young SP. (*M* = 15 years; *SD* = 1.1)	Youth players completed two test sessions (control vs. MF) using a crossover design. MF was induced by 30 min of smartphone puzzle gameplay (“Brain It On” app). Physical performance (Yo-Yo IR1) and technical skill (LSPT) were assessed under both conditions.	“Brain It On” app and RPE	Yo-Yo IRT1 and LSPT.	Prolonged smartphone use induced MF and negatively affected the physical and technical performance of young footballers.
Kunrath et al. (37)	How does MF affect soccer performance during SSG? A cognitive, tactical and physical approach.	To investigate the impact of MF on the peripheral vision, tactical decision-making, and physical capabilities of SP while they engage in a controlled SSG.	18 male university first-team SP. (*M* = 21.8 years; *SD* = 2.5)	SP completed two SSG sessions (“Goalkeeper+3 *vs.* 3 + Goalkeeper”) under control (documentary) and MF (30 min ST) conditions. MF (VAS), peripheral perception (VTS), and tactical behavior (FUT-SAT) were assessed. GPS data were collected to monitor physical performance.	Modified ST; VAS.	VTS; FUT-SAT and GPS.	MF reduced perception and tactical accuracy, while players covered greater total distance and moderate-speed running distance, suggesting compensatory physical effort during SSG.
Nishida et al. (33)	Daytime napping benefits passing performance and scanning activity in elite SP.	To investigate how daytime napping influences the scanning behavior of elite collegiate SP.	14 male elite collegiate SP. (*M* = 21.6 years; *SD* = 0.5)	The study had a crossover design with SP randomized into nap and no-nap conditions. The nap group had the chance to take a 40 min nap; the no-nap group did not. Cognitive function was assessed via the TMT, and passing performance and scanning activity were evaluated via a modified LSPT.	KSS and VAS for sleepiness/perceived fatigue.	TMT, LSPT and Scanning Activity.	Daytime napping improved passing-test performance time and increased scanning activity, suggesting that recovery-related cognitive states may influence information acquisition and technical execution in football-specific passing tasks.
Rubio-Morales et al. (42)	The mental fatigue induced by physical, cognitive and combined effort in amateur soccer players: A comparative study using EEG	To analyze the subjective, behavioral and neural effects of cognitive, physical and combined tasks on mental fatigue in amateur soccer players.	13 amateur soccer players; 10 males and 3 females (M = 23 years; SD = 5.43)	Randomized crossover repeated-measures design with three 30-min conditions: cognitive task, physical task and combined cognitive-physical task. The cognitive protocol consisted of a 30-min incongruent ST; the physical protocol consisted of 30 min cycling at 65–75& of an estimated peak heart rate; the combined protocol consisted of cycling at the same intensity while Simultaneously performing the ST.	VAS; perceived cognitive load; EEG measures including IAPF, alpha and theta midline power.	PVT-B reaction time and TacticUP soccer-specific decision-making test.	Cognitive and combined protocols produced greater perceived MF than physical exercise alone. Psychomotor reaction time worsened after cognitive and combined conditions. EEG showed reduced IAPF after the cognitive protocol and increased alpha power in frontal/parietal midline regions. Soccer-specific decision-making was not significantly affected.
Silva et al. (38)	Does experience mitigate the deleterious effect of mental fatigue on tactical performance? A study in youth soccer academies	To examine the effects of MF or cognitive-load conditions on collective behavior, spatial organization, or positional dynamics in football.	Young male football players.	Football-specific task or SSGs condition under MF/cognitive-load manipulation.	MF or cognitive-load assessment through subjective and/or task-based measures.	GPS-derived, positional, spatial-dispersion, synchronization, or collective-organization variables.	MF or cognitive-load conditions affected collective spatial behavior and/or positional organization, suggesting that cognitive strain may influence team coordination and tactical structure.
Skoki et al. (23)	Exploring the impact of the perceived cognitive load on the physical performance in soccer.	To explore how perceived cognitive load relates to various aspects of physical performance; and to assess different motivation profiles in SP, by utilizing a machine learning approach.	16 players initially; goalkeepers excluded from external-load analysis; combined NASA-TLX/GPS analysis included 14 players and 63 responses. (*M* = 23 years; *SD* = 4)	SP were monitored over 19 sessions using GPS and NASA-TLX. They were grouped by perceived cognitive load (Z-scores) and motivation (K-means). Physical performance (e.g., total distance, high-speed running) was compared across groups.	NASA-TLX.	GPS.	Higher perceived cognitive load was associated with changes in GPS-derived performance outcomes, suggesting that cognitive-load states may influence external-load expression and collective behavior during football-specific tasks.
Smith et al. (29)	MF impairs soccer-specific physical and technical performance.	To examine how MF influences both the physical and technical performance specific to soccer.	Two studies: 12 moderately trained players for Yo-Yo IR1; 14 experienced players for LSPT/LSST. (*M* = 21.5 years; *SD* = 3.0)	In two crossover trials, players completed the Yo-Yo IR1, LSPT, and LSST following either 30 min of ST (MF) or control (magazine reading). MF, effort, and motivation were measured via VAS. Physical (distance, HR, RPE) and technical performance (penalties, shot speed/accuracy) were assessed.	VAS and RPE.	Yo-Yo IR1, HR, LSPT and LSST.	MF negatively impacts soccer-specific performance, impairing intermittent running performance, passing accuracy, and shooting performance. This study highlights the importance of managing MF in soccer to maintain optimal performance levels.
Smith et al. (40)	MF impairs soccer-specific decision-making skill.	To explore the impact of artificially induced MF on decision-making abilities specific to soccer skills.	12 well-trained male SP. (*M* = 19.3 years; *SD* = 1.5)	SP performed a DMT twice (control vs. 30 min ST). MF, effort, and motivation were assessed via VAS. Decision-making was evaluated using response accuracy, response time, and ETS for VS.	VAS, DMT and ETS.	Soccer-specific film-based decision-making task; response accuracy, response time, and VS.	MF impaired soccer-specific decision-making by decreasing response accuracy and increasing response time, while most VS variables showed unclear effects.
Smith et al. (19)	Impact of MF on speed and accuracy components of soccer-specific skills.	To investigate how MF affects the speed and accuracy aspects of soccer-specific skills	14 experienced male SP. (*M* = 19.6 years; *SD* = 3.5)	SP performed the LSPT twice (control vs. 30 min ST), 48 h apart. MF, effort, and motivation were assessed via VAS. Passing accuracy and speed were analyzed across conditions.	VAS.	LSPT.	MF negatively affects soccer-specific skills, particularly accuracy in passing. While movement speed remained unchanged, players exhibited decreased performance in terms of missed targets and perfect passes when mentally fatigued. This suggests that managing MF is essential for maintaining optimal performance in soccer.
Soltani et al. (34)	Comparing the effect of mental fatigue-inducing models on selected cognitive and technical performance aspects in young soccer players.	To compare four MF-inducing models and identify which protocol most effectively induces MF and affects cognitive and technical performance in young soccer players.	15 male young soccer players, aged 16–18 years, with competitive experience,	Randomized counterbalanced crossover design with four 30-min protocols: Modified Stroop, SAFT90, T-SAFT90, and combined T-SAFT90 + Stroop. Pre/post assessment were conducted in each condition.	VAS; ST; Captain's Log cognitive tasks including working memory capacity, visual scanning identification and auditory pattern recognation.	LSPT outcomes: penalty time, movement time and Passing accuracy; HR and RPE/motivation monitored as contextual variables.	All protocols increases perceived mental fatigue, with the combined T-SAFT90 + Stroop protocol producing the greatest increase. Cognitive-loaded protocols impaired response accuracy/time and several cognitive domains. Technical performance was negatively affected, particularly penalty time, movement time and Passing accuracy. The combined cognitive-physical model appears the strongest acute MF-induction protocol, Although ecological validation in real soccer contexts remains necessary
Soylu and Arslan (24)	Effects of MF on psychophysiological, cognitive responses, and technical skills in SSG in amateur SP.	To evaluate how MF influences the psychophysiological and cognitive responses, as well as technical skills, during SSG among amateur SP.	18 male amateur SP. (*M* = 19.1; *SD* = 1.2)	SP performed 2 *vs.* 2, 3 *vs.* 3, and 4 *vs.* 4 SSG under two conditions: with and without MF (30 min ST). Psychophysiological (VAS-A, FS, FAS, MTV), cognitive (TMT), and technical performance (via video analysis) were assessed post SSG.	ST, FS, FAS, VAS-A, LS and TMT.	TMT and technical actions during SSG.	MF negatively affected psychophysiological and cognitive responses and impaired several technical skills during SSGs, particularly in smaller game formats (2v2 and 3v3). These findings suggest that coaches should consider players’ mental state when designing SSG formats.
Soylu et al. (25)	Effects of MF on the psychophysiological responses, kinematic profiles, and technical performance in different SSG.	To evaluate how MF impacts the psychophysiological responses, movement patterns, and technical performance of young soccer players engaged in SSG.	24 young male SP. (*M* = 15.9 years; *SD* = 1)	SP played 2 *vs.* 2, 3 *vs.* 3, and 4 *vs.* 4 SSG with and without prior MF (30 min ST). HR, distance, RPE, VAS, RSME, PACES, BRUMS, and technical performance (passes, shots, interceptions, lost balls) were assessed across formats.	ST, BRUMS, RPE, RSME, FS and PACES.	HR, total distance covered, and technical actions during SSG.	MF negatively affected psychophysiological responses, mood, technical performance, and movement demands during SSGs in young soccer players. Players under MF conditions covered less distance and showed poorer technical performance, including more lost balls and unsuccessful passes.
Soylu et al. (26)	Effects of mental fatigue on psychophysiological responses, kinematic variables and technical actions in small-sided soccer games: a time course analysis	To examine MF-related responses during football-specific or SSGs performance.	Male SP.	Football-specific task or SSGs condition under MF/cognitive-load manipulation.	Subjective MF, mental effort, perceived exertion and/or psychophysiological responses	Technical, physical, tactical and/or SSG-related performance variables.	MF-related responses were associated with changes in football-specific performance, particularly in psychophysiological, technical, or external-load outcomes depending on the task format.
Staiano et al. (28)	BET Improves Physical, Cognitive, and Multi-tasking Performance in Professional Football Players	To assess the impacts of post-BET during the preseason training phase of professional SP; to their knowledge, this is the inaugural research examining the effects of BET on SP.	25 male professional SP (22 completed the study). (*M* = 22.4 years; *SD* = 4.3)	Players completed a 4-week preseason training program. The BET group performed cognitively demanding tasks (flanker, go/no-go, AX-CPT) after physical training sessions, whereas the control group listened to neutral sounds. Physical and cognitive performance were assessed pre- and post-intervention.	ST, NASA-TLX and cognitively demanding BET tasks.	30–15 IFT; repeated sprint ability random test; soccer-specific reactive agility test; PVT; FITLIGHT.	Integrating BET into preseason training for professional football players is more effective than standard physical training alone. BET improved intermittent fitness, reactive agility, repeated-sprint performance, ST performance, and psychomotor vigilance compared with standard physical training alone.

In the “MF” column, the term refers broadly to mental-fatigue induction procedures, mental-fatigue assessment tools, or closely related fatigue/cognitive-load measures, depending on the design of each study. Therefore, not all studies included an experimental MF induction protocol. SP, soccer players; M, mean; SD, standard deviation; ST, stroop task; RA, response accuracy; RSME, rating scale of mental effort; RPE, rating of perceived exertion; VS, visual search behaviors; HR, heart rate; RSA, repeated-sprint ability; DV, documentary viewing; PVT, psychomotor vigilance test; NIRS, near-infrared spectroscopy; NASA-TLX, NASA task load index; BL, blood lactate; VAS, visual analogue scale; Yo-Yo IR1, Yo-Yo intermittent recovery test level 1; SSG, small-sided games; GPS, global positioning system; CMJ, countermovement jump; JA, jump assessments; RCOD, repeated change of direction task; FUT-SAT, system of tactical assessment in soccer; SSA, software soccer analyzer; LSPT, loughborough soccer passing Test; LSST, Loughborough soccer shooting test; DMI, decision-making index; GPAI, game performance assessment instrument; TQR, total quality of recovery scale; UCI, urine color index; HSM, heart stress monitor; SCMS, simulated congested match schedule; PDM, passing decision-making; VTS, Vienna test system; MFI-20, multidimensional fatigue inventory-20 items; GF, general fatigue; PF, physical fatigue; RedAct, reduced activity; RM, reduced motivation; ABQ, athlete burnout questionnaire; FL/KF, time from landing of the support foot to maximum knee flexion; FL/BC, time from landing of the support foot to ball contact; FPRON, time from landing of support foot to maximum foot inversion; TCT, total contact time; HRV, heart rate variability; RMSSD, root mean square of successive differences; SDSD, standard deviation of successive differences; SDNN, standard deviation of normal-to-normal intervals; LFnorm, normalized low-frequency power; HFnorm, normalized high-frequency power; LF/HF, low-frequency/high-frequency ratio; TMT, trail making test; KSS, Karolinska sleepiness scale; IAPF, individual alpha peak frequency; PVT-B, brief psychomotor vigilance test; DMT, decision-making task; ETS, eye-tracking system; FS, feeling scale; FAS, felt arousal scale; VAS-A, visual analogue scale - anxiety; LS, likert scale; PACES, physical activity enjoyment scale; BRUMS, Brunel mood scale; BET, brain endurance training; IFT, intermittent fitness test; FITLIGHT, FitLight trainer system; SAFT90, soccer-specific aerobic field test 90; T-SAFT90, technical soccer-specific aerobic field test 90.

### 2.5 Analyzing studies and extracting data

In collaboration with the other authors, a systematic, standardized approach was used to extract data from the selected studies, which ensured consistency in the information extraction and analysis. A summary database (Table 2) was constructed to report the main features of the included studies (reference, title, objectives, participants, intervention, instruments or variables, and main results and conclusions). In order to give a full description of their methods, the instruments used to assess MF and a measure of performance were also noted. Each study was examined in detail, paying particular attention to the methodological design and target population. The analysis considered the research design of each study in order to provide a clearer overview of the different methodological approach-es to examining MF in football. Through this consideration, it allowed for an understanding of the different research approaches taken to investigate the concept of MF within the sport of football. Regarding the study participants, we extracted detailed information, including their age group (youth or adult), gender, competitive level (professional or amateur players), and sample size. This provided a means for a comprehensive overview of the populations studied, identifying possible gaps in the literature based on availability of data from underrepresented groups or levels of play.

In order to give a more in-depth examination of the instruments utilized, the column regarding “instruments and variables” was separated into two subcategories (“MF” and “Performance”). The distinction was made in order to differentiate tools/methods used for measuring MF and tools/methods used in assessing performance with specific reference to football. This aided comparability across studies, allowing determination of the most commonly adopted methodologies and potential gaps in the literature. data were extracted independently by three reviewers for each article using this predefined form. If there were discrepancies, a fourth review was included to reach a consensus. This approach enabled data reliability and reduced potential biases. The extracted data were then described analytically, providing the synthesis of methodological approaches and the principal key findings pertinent to the objectives of the study. The emergence of “main results and conclusions” category offered insight, allowing readers to more readily absorb the key takeaways and conclusions from study to study and better appreciate the weight of the cumulative evidence. Due to heterogeneity in MF induction protocols, participant characteristics, study designs, assessment tools, and performance outcomes, a quantitative meta-analysis was not considered methodologically appropriate; therefore, a qualitative systematic synthesis was conducted.

## 3 Results

A total of 668 records were identified across the five databases. After removal of 34 duplicates, 634 records were screened by title and abstract. Of these, 455 records were excluded according to the primary reason for exclusion: not related to MF or cognitive fatigue (*n* = 164), not football/soccer-specific context (*n* = 118), no relevant performance/monitoring outcome (*n* = 66), ineligible publication type (*n* = 70), and non-empirical or insufficiently relevant design (*n* = 37). The remaining 179 reports were sought for retrieval and assessed for eligibility. Of these, 151 reports were excluded with reasons: review articles (*n* = 20), non-football sport context (*n* = 12), not related to MF and football performance (*n* = 88), opinion/editorial articles (*n* = 6), outside publication date range (*n* = 19), and ineligible participant population (*n* = 6). Finally, 28 studies met all inclusion criteria and were included in the systematic review, as shown in Figure 1.

Inter-rater reliability (IRR) is a crucial aspect of systematic review that helps validate studies by measuring the degree to which different researchers agree on which studies to include and how to analyze them. IRR is frequently assessed by Cohen's kappa coefficient (12), a statistic that quantifies the degree of agreement between two or more reviewers, thereby ensuring the impartiality and soundness of the review outcomes. Cohen's kappa coefficient was computed (13) to maintain the consistency and objectivity of the article selection process, as it measures the degree of agreement between the three reviewers (the first author and two external researchers). There were no discrepancies between the three researchers for the entire analysis, including all selected 28 articles using the MMAT; therefore, Cohen's kappa coefficient was 1, which indicates perfect agreement (14) of evaluators in studies analysis.

### 3.1 Impact of MF on football performance

The first objective of this systematic review was to understand how MF affects performance in football. Based on the 28 studies analyzed, the evidence was organized into four domains: physical, technical, tactical and cognitive performance. Importantly, findings were interpreted by distinguishing between objective performance outcomes, subjective psychophysiological responses, mixed findings, and non-significant results, rather than relying exclusively on the number of studies reporting an effect.

#### 3.1.1 Physical performance

Evidence regarding the effects of MF on physical performance was mixed and depended strongly on the type of outcome assessed. Several studies reported objective decrements in physical output, particularly in endurance-related, high-intensity running, or small-sided-game variables. For example, Filipas et al. (15), Greco et al. (16), and Smith et al. (2016) used the Yo-Yo IR1 and reported reductions in total distance covered or aerobic performance following MF induction.

By contrast, sprint-based, RSA, GPS-derived, and small-sided-game showed fewer uniform effects. Some studies reported stable sprint or repeated-sprint performance despite increased perceived effort or psychophysiological strain, particularly in RSA or soccer-specific performance contexts (17–19) GPS-based and SSG studies further indicated that fatigue-related conditions may alter running profiles, external load, and collective movement patterns, although not all changes in high-intensity activity were attributable specifically to MF (20–26).

Additional evidence from monitoring or applied-performance contexts suggests that MF-related responses may interact with training load, recovery status, and match demands over time (27). Staiano et al. (28) provides indirect evidence that improving tolerance to MF through brain endurance training may benefit repeated-sprint or agility-related outcomes, but this should be distinguished from studies examining the acute detrimental effects of MF.

Therefore, the available evidence suggests that MF may influence physical performance through two partially distinct pathways: measurable reductions in objective physical output in some contexts, and increased perceived effort or psychophysiological strain in others. These outcomes should not be interpreted as equivalent. Rather, the evidence indicates that MF can increase the subjective cost of performance even when external physical metrics remain unchanged. The strength and consistency of evidence are greater for increased perceived exertion and mental effort than for uniform impairments in objective physical-output measures, which appear to vary according to sample characteristics, task design, and performance assessment method.

#### 3.1.2 Technical performance

At the technical level, several studies reported impairments in football-specific skill execution under MF conditions, particularly in passing and shooting tasks. However, the consistency of these effects varied according to the type of technical outcome assessed, with some studies showing clear reductions in accuracy or increased errors, while others suggested more subtle changes in decision quality or execution efficiency. These studies suggest that MF is associated with selective impairments in technical decision-making and motor execution, particularly in tasks requiring accuracy, speed, or inhibition under cognitive load.

Passing performance was frequently assessed through the LSPT or football-specific passing tasks. Smith et al. (29) reported impairments in passing-related outcomes following MF induction, including longer execution or penalty times, increased errors, or reduced overall technical efficiency. Smith et al. (19) also reported decrements in soccer-specific performance under MF, although the exact technical outcomes should be described according to the specific test used in that study. Similarly, Filipas et al. (15) and Greco et al. (16) reported that passing performance was compromised following MF induction, particularly in youth players, with effects on execution time, errors, or overall LSPT performance.

Shooting-related outcomes were assessed mainly through the LSST or penalty-kick tasks. Smith et al. (29) reported poorer shooting outcomes under MF using the LSST. Technical performance during small-sided games was also affected in several studies. Badin et al. (18) reported impaired technical performance during SSGs under MF conditions, while Soylu and Arslan (24), Soylu et al. (25), and Soylu et al. (26) reported reductions in technical indicators or technical fluency across different SSG formats or cognitive-load conditions.

In decision-making performance studies, Fortes et al. (30), Fortes et al. (31), and Gantois et al. (32) observed that MF induced by smartphone use, video games, or Stroop tasks impaired passing decision-making accuracy or efficiency within simulated match scenarios. Importantly, these impairments did not always correspond to a lower number of passes completed, suggesting that MF may affect the quality of technical decisions more than the quantity of technical actions. Nishida et al. (33) added a recovery-related perspective by showing that daytime napping benefited passing performance and scanning activity, suggesting that cognitive recovery may support technical execution through improved information acquisition.

More recently, Soltani et al. (34) further showed that different MF-inducing models altered LSPT-related technical outcomes, with the combined T-SAFT90 + Stroop protocol producing the greatest subjective MF and notable decrements in passing-related performance. Together, these findings indicate that MF can impair technical performance, especially when tasks require precision, rapid information processing, and accurate motor execution under cognitive load.

#### 3.1.3 Tactical performance

In relation to the tactical dimension, several studies investigated the effects of MF on players’ collective behavior, decision-making, positioning, and tactical coordination on the pitch. Overall, these studies suggest that MF may negatively influence players’ ability to adapt to dynamic game situations, although the strength of evidence varies across tactical assessment tools and study designs.

Studies using FUT-SAT or tactical-principle-based assessment showed that MF can reduce the quality or frequency of tactical actions and impair adherence to offensive and defensive tactical principles. Da Silva et al. (35), da Silva et al. (36) and Kunrath et al. (37) observed a reductions in the number or quality of offensive and defensive tactical actions under MF, with players demonstrating poorer adherence to core tactical principles such as penetration, coverage, or support.

Collective behavior synchronization, and spatial organization were mainly addressed in SSG or positional-tracking studies. Coutinho et al. (20) found that MF and additional spatial references affected players’ physical and tactical performances during SSGs, suggesting that MF can disrupt the use of environmental information and tactical positioning. Coutinho et al. (21) extended these findings by showing that fatigue-related conditions altered collective behavior, including dyadic distances, synchronization, and team spatial organization. Positional and GPS-derived evidence also supports this interpretation, with Skoki et al. (23) and Silva et al. (38) showing that cognitive load or MF-related conditions can affect spatial organization, synchronization, and collective movement behavior.

In studies focusing more on anticipation, tactical judgement, or decision-making as a tactical behavior, Alder et al. (39), Smith et al. (40), and Fortes et al. (41) support the interpretation that MF may impair players’ ability to anticipate play, select context-appropriate actions, or make tactical decisions under cognitively demanding conditions. Donnan et al. (22) extended this evidence to women's football, showing that MF altered attentional focus, communication patterns, and psychological responses during small-sided games, even when external work output was not significantly affected.

These findings suggest that MF may undermine not only individual execution but also the collective dimension of football performance, by reducing tactical adaptability, positioning accuracy, and responsiveness to situational demands. These impairments may be especially relevant in team sports, where tactical coordination is fundamental for success.

#### 3.1.4 Cognitive performance

In the cognitive domain, several studies examined how MF influences psychological mechanisms involved in attention, information processing, and decision-making efficiency. The evidence was generally consistent in showing slower responses, reduced attentional efficiency, or impaired decision-making accuracy, although the magnitude and statistical robustness of these effects varied across computerized tasks, video-based decision tasks, neurophysiological measures, and football-specific assessments.

Vigilance, sustained attention, and reaction-time outcomes were assessed using tools such as the PVT or similar cognitive tasks. Angius et al. (17) applied the PVT and observed slower reaction times or reduced attentional capacity following MF induction, despite stable physical output. Beyond acute PVT-based impairments, Staiano et al. (28) showed that brain endurance training improved cognitive and multi-tasking performance, supporting the relevance of sustained-attention capacity for football performance under fatigue. Rubio-Morales et al. (42) further extended this evidence by showing that cognitive and combined fatigue protocols worsened psychomotor performance and altered neurocognitive responses, reinforcing the link between MF, vigilance, and reaction-time efficiency.

Anticipation, visual search, and information acquisition were addressed in studies examining perceptual-cognitive behavior. Alder et al. (39) demonstrated that mental load impaired skilled players’ anticipatory judgements, particularly when combined with physical load, indicating that MF can compromise perceptual decision-making in representative football scenarios. Nishida et al. (33) added a recovery-related perspective by showing that daytime napping benefited passing performance and scanning activity, suggesting that cognitive recovery may support technical execution through improved information acquisition.

Cognitive flexibility, working memory, response accuracy, and neurophysiological indicators were assessed in more recent studies using cognitive batteries or neurophysiological tools. Neurophysiological and cognitive-test evidence further supports the sensitivity of cognitive outcomes to MF. Rubio-Morales et al. (42) reported neurocognitive changes following cognitive and combined fatigue protocols while Soltani et al. (34) extended these findings using a broader cognitive battery, showing that different MF-inducing models affected response accuracy, response time, working memory, visual scanning, and auditory pattern recognition. In parallel, Soylu and Arslan (24) and Skoki et al. (23) used workload- and attention-related measures such as NASA-TLX, TMT, or perceived cognitive load, showing that MF can increase mental effort and cognitive workload alongside changes in football-specific performance.

Taken together, these studies suggest that MF can disrupt the perception-decision-action loop fundamental to high-performance football, particularly by overloading attentional resources and slowing down response processes. These effects may reduce individual performance efficiency and impair players’ ability to adapt to complex tactical demands under pressure.

### 3.2 Participant characteristics: gender and competitive level

The included studies predominantly assessed male football players, revealing a substantial gender imbalance in the current research landscape on MF in football. However, this pattern was not absolute, as Donnan et al. (22) examined women's football players, providing one of the few examples of MF research conducted in female football population and highlighting the limited representation of women in this literature. This limited representation of female participants restricts the generalizability of the findings and highlights a critical gap in the literature, particularly given the growing professionalism and performance demands in women's football.

In terms of competitive level, the included studies covered professional, elite, youth, collegiate, amateur, and applied monitoring contexts. Professional or elite-level players were examined in studies such as Angius et al. (17), Badin et al. (18), Fortes et al. (31), Fortes et al. (41), Gantois et al. (32), Smith et al. (29), Smith et al. (40), and Smith et al. (19). These studies often included players with higher training loads and competitive exposure, which may contribute to greater psychological resilience and more refined cognitive-tactical strategies under mental stress. From an applied monitoring perspective, Brett et al. (27) contributed evidence by examining MF-related responses across an extended competitive period, supporting the relevance of considering fatigue beyond isolated laboratory protocols.

Several studies examined youth, academy, or young competitive players, a group that may be more vulnerable to the effects of MF due to ongoing neurocognitive development and limited tactical experience. These include Coutinho et al. (20), Coutinho et al. (21), da Silva et al. (35), da Silva et al. (36), Greco et al. (16), Filipas et al. (15), Silva et al. (38), and Soltani et al. (34). Soltani et al. (34), for instance, examined male players aged 16–18 and found that different MF-inducing models increased subjective MF and altered cognitive and technical performance.

Evidence from amateur, collegiate, and lower-level football samples was provided by Kunrath et al. (37), Rubio-Morales et al. (42), and Soylu and Arslan (24), indicating that MF-related impairments are not restricted to elite or professional players but also emerge in less specialized or developmentally diverse competitive contexts. Findings in these contexts were generally negative but may be less predictable due to greater inter-individual variability in cognitive load management, training background, and match experience.

This demographic imbalance, favoring male athletes and particularly trained or competitive samples, limits the applicability of the findings to broader football populations. Future studies should incorporate female athletes and a wider spectrum of competitive levels, from grassroots to professional, in order to better capture the full scope of MF in football. In summary the literature generally indicates that MF can negatively affect physical, technical, tactical, and cognitive components of football performance, with the magnitude of its impact varying according to age, sex, competitive level, task type, and assessment method.

### 3.3 Methodologies used to identify and analyze MF in football

The second aim of this systematic review was to analyze the methods used to induce and measure MF in the football-specific context. The literature reflects a marked preference for experimental protocols, often conducted under laboratory or semi-controlled conditions, in which MF is induced through cognitive-load tasks, before participants complete physical, technical, tactical, or cognitive performance assessments.

The Stroop Test was the most frequently used MF induction method, appearing either as a standalone protocol or as part of combined cognitive-physical protocols. Stroop-based or modified Stroop protocols were used in studies assessing physical, technical, tactical, or cognitive outcomes, including Angius et al. (17), Badin et al. (18), Gantois et al. (32), Kunrath et al. (37), Rubio-Morales et al. (42), Soltani et al. (34), Smith et al. (29), and Smith et al. (40). This task is commonly used because it challenges selective attention and response inhibition. However, the ecological validity of laboratory-based cognitive tasks, such as the Stroop task, remains a methodological limitation, as these protocols do not fully reproduce the perceptual, interactive, and decision-making demands of football-specific performance (20, 21, 24, 31, 34, 41).

Other studies induced MF through smartphone use, social-media exposure, or video-game protocols. Smartphone- or social-media-based MF protocols were used in Donnan et al. (22), Fortes et al. (30), and Fortes et al. (31), while video-game-induced MF was used in Fortes et al. (41). These approached may offer greater ecological relevance than purely laboratory-based cognitive tasks, particularly because they reflect common pre-match or daily-life behaviors among football players.

Some studies used football-specific, repeated-match, or combined cognitive physical protocols. Coutinho et al. (20) and Coutinho et al. (21) examined fatigue-related effects in SSG or match-like contexts, while Staiano et al. (28) investigated brain endurance training as an approach to improving tolerance to MF. Soltani et al. (34) compared Modified Stroop, SAFT90, T-SAFT90, and a combined T-SAFT90 + Stroop protocol, showing that all protocols increased subjective MF, with the combined cognitive-physical model producing the largest effect.

Regarding MF assessment, subjective instruments were the most common. Self-reported MF, motivation, and mental effort were frequently measured using the VAS, while perceived workload was assessed in some studies using the NASA-TLX. These subjective or workload-based approaches were particularly relevant in studies such as Rubio-Morales et al. (42), Skoki et al. (23), Soylu and Arslan (24), and Soylu et al. (25). Although these tools provide practical information on athletes’ perceived fatigue states, their subjective nature raises questions about standardization and validity, particularly when administered in isolation.

Objective, physiological, or neurophysiological assessments were less frequently used than subjective scales, but included HRV, NIRS, PVT, eye-tracking, and movement-timing analyses. Angius et al. (17) combined NIRS and PVT-related outcomes to examine cerebral oxygenation and sustained-attention responses under MF, while Staiano et al. (28) incorporated PVT-related assessment in the context of brain endurance training. More recent studies broadened this approach by including neurophysiological, psychophysiological, and movement-based measures: Rubio-Morales et al. (42) contributed evidence from EEG-, ERP-, or neurocognitive assessments.

Regarding performance evaluations, several studies implemented standardized physical tests such as the Yo-Yo IR1, 30-15 IFT, RSA protocols, GPS-derived variables, and SSG-based external-load measures. Yo-Yo IR1 outcomes were assessed in Filipas et al. (15), Greco et al. (16), and Smith et al. (29), while RSA or sprint-related outcomes were examined in Angius et al. (17), Badin et al. (18), Skoki et al. (23) and Smith et al. (19). GPS-derived or SSG-based external-load measures were used in Coutinho et al. (20), Coutinho et al. (21), Donnan et al. (22), Silva et al. (38), Soylu and Arslan (24), and Soylu et al. (26).

In terms of technical performance, the LSPT was used in several studies to assess passing performance, including Filipas et al. (15), Greco et al. (16), Soltani et al. (34), and Smith et al. (29). The LSST was used by Smith et al. (29) to assess shooting performance. Other studies used football-specific drills, simulated match scenarios, SSGs, or penalty-kick tasks to assess passing, shooting, dribbling, and decision-making performance, including Badin et al. (18), Fortes et al. (30), Fortes et al. (31), Fortes et al. (41), Gantois et al. (32), Nishida et al. (33), Soylu and Arslan (24), Soylu et al. (25), and Soylu et al. (26).

At the tactical level, validated instruments and positional-tracking approaches were used to measure collective performance under MF. FUT-SAT-based analyzes of tactical principles were used in da Silva et al. (35), da Silva et al. (36), and Kunrath et al. (37). GPS- or positional-derived measures of inter-player distance, team synchronization, spatial dispersion, and collective organization were used in Coutinho et al. (20), Coutinho et al. (21), Silva et al. (38), and Skoki et al. (23).

Several studies assessed cognitive performance under MF, covering reaction time, attentional control, response accuracy, working memory, visual scanning, anticipation, vigilance and decision-making speed. Tools included Stroop-based cognitive tests, PVT, TMT, NASA-TLX, video-based decision-making tasks, cognitive software batteries, and neurophysiological measures. Decision-making tasks were used in Fortes et al. (30), Fortes et al. (31), Fortes et al. (41), Gantois et al. (32), and Smith et al. (40). Anticipation and visual-search-related outcomes were addressed in Alder et al. (39) and Nishida et al. (33). Cognitive-test batteries or neurophysiological outcomes were used in Rubio-Morales et al. (42), and Soltani et al. (34).

Overall, the methodological heterogeneity identified across studies reflects a prevailing lack of standardization regarding MF operationalization and measurement in football. Although recent studies have introduced more ecologically valid and football-specific approaches, many protocols still rely on laboratory-based cognitive tasks that may not fully transfer to on-field performance. Future studies should prioritize mixed-method designs that combine subjective, physiological, cognitive, and football-specific performance assessments in ecologically valid settings, such as training camps or match-like situations.

## 4 Discussion

This systematic review synthesized current evidence on the effects of MF on football performance, based on 28 empirical studies published between 2014 and 2026. The findings indicate that scientific interest in this topic has increased substantially, particularly in recent years; however, the field remains fragmented, with important gaps related to methodological consistency, ecological validity, longitudinal monitoring, and population diversity. This fragmentation limits the comparability of findings across studies and constrains the development of practical, evidence-based monitoring protocols for football-specific contexts.

A clear pattern across the included studies is the continued reliance on controlled experimental protocols, frequently using isolated cognitive tasks such as the Stroop task to induce MF. These approaches provide high levels of internal validity by controlling potential confounding variables and strengthening causal inference regarding the effects of MF on football performance. However, their ecological validity remains limited because isolated laboratory tasks do not fully reproduce the perceptual, interactive, tactical, and contextual demands of football-specific performance. This limitation has been addressed directly or indirectly by studies using small sided games, football-specific decision-making tasks, match-like simulations, smartphone or video-game protocols, and combined cognitive-physical models (20, 21, 24, 31, 34, 41). Consequently, an important challenge for future research is to balance experimental control with ecological representativeness in football-specific contexts.

Similarly, subjective assessments, particularly VAS, RPE and NASA-TLX, were widely used due to their practicality and ease of implementation in applied settings. However, the predominance of subjective tools also limits standardization, especially when they are used without complementary objective indicators. Several studies attempted to address this limitation by incorporating objective, physiological, neurophysiological, or behavioral measures, including PVT, NIRS, HRV, EEG/ERP, eye-tracking, GPS-derived variables, and movement-timing analysis (17, 23, 28, 42). Nevertheless, the use of these instruments in routine training environments remains constrained by logistical, financial, and technical barriers. The absence of validated, field-based measurement tools therefore represents a critical gap between research and practice. In addition, some included studies investigated constructs conceptually related to MF, such as perceived cognitive load, recovery-related cognitive fatigue, or sleep-related cognitive impairment, or chronic fatigue-related responses, rather than experimentally induced MF directly. These studies were retained because they provided relevant indirect evidence regarding cognitive strain and its potential influence on football performance. Nevertheless, this conceptual heterogeneity reinforces the need for clearer operational definitions and standardized assessment criteria in future research.

Another recurring limitation concerns the relatively limited number of studies examining the effects of MF on collective tactical dynamics. Although the evidence has expanded beyond isolated physical and technical outcomes, tactical performance remains less frequently assessed than subjective fatigue, physical output, or individual technical execution. Studies using FUT-SAT, positional tracking, GPS-derived variables, and small-sided game designs suggest that MF may impair tactical principles, collective behavior, spatial organization, synchronization, and decision-making under representative playing conditions (20, 21, 23, 35–38). Nevertheless, most studies still examine performance domains separately rather than modeling the integrated physical, technical, tactical, and cognitive demands of match play. This methodological fragmentation undermines efforts to develop holistic monitoring frameworks capable of capturing the multidimensional nature of performance decrements driven by MF.

The lack of sample diversity is also a critical limitation. The evidence base is still predominantly composed of male football players, often from professional, elite, youth academy, collegiate, or amateur contexts. However, the previous absence of female participants is no longer absolute, as Donnan et al. (22) examined women's football players, providing an important but still isolated contribution to this literature. Despite this exception, female football remains markedly underrepresented, limiting the external validity and practical applicability of current findings. Evidence from amateur, collegiate, and lower-level samples has increased in recent studies, but remains less extensive than evidence from trained or competitive male cohorts (24, 37, 42). This sampling imbalance constrains the generalizability of current evidence and limits the development of population-specific monitoring protocols. Future research should therefore include more female players and a wider range of competitive levels, age groups, and developmental contexts.

Longitudinal research on MF in football remains scarce. Most included studies examined the acute effects of experimentally induced MF, whereas fewer studies considered how MF-related responses may accumulate, fluctuate, or dissipate across training microcycles, congested schedules, recovery periods, or extended competitive contexts. Recent applied or monitoring-oriented studies have begun to address this gap by considering MF-related responses in more ecologically relevant or extended settings (22, 27). Nevertheless, the field still lacks robust longitudinal designs capable of determining how MF evolves across the competitive season and how it interacts with match congestion, travel, recovery quality, academic or occupational demands, and extra-sporting stressors. This limits the practical utility of current evidence for season-long monitoring and management strategies.

Finally, few studies have adopted multidimensional performance assessment models. Although several investigations assessed more than one performance domain, most studies still emphasized physical, technical, tactical or cognitive components in isolation. This approach creates artificial distinctions between domains that are highly interdependent during match play. Studies combining decision-making, technical execution, workload, GPS-derived variables, or tactical outcomes provide important progress toward more integrated assessment models (23, 24, 31, 34, 38). However, the field still lacks standardized frameworks capable of simultaneously capturing the physical, technical, tactical, cognitive, perceptual, and psychophysiological consequences of MF in representative football contexts. This limitation hinders the development of comprehensive monitoring systems for applied football environments.

Overall, this review confirms that MF research in football is developing rapidly but remains methodologically and conceptually fragmented. The field currently lacks sufficient methodological integration, ecological representativeness, longitudinal evidence, and population coverage. Despite these limitations, the growing number of recent studies indicates a clear progression toward more applied, multidimensional, and football-specific research designs. To ensure meaningful progress, future research should prioritize methodological consensus on:

- Standardized protocols for inducing ecologically valid MF.
- Comprehensive assessment batteries combining subjective, behavioral, physiological, and neurophysiological measures.
- Domain-spanning performance evaluation frameworks capable of capturing the multi-dimensional and integrated nature of football performance.
- Longitudinal monitoring models capable of tracking acute and chronic MF across training and competition periods.

### 4.1 Gaps in the literature and future recommendations

Despite the recent increase in studies on MF in football, this review identified significant gaps in the literature. Existing research remains concentrated predominantly on male players and trained competitive samples, although recent evidence has begun to include female, collegiate, amateur, and applied monitoring contexts. Nevertheless, female athletes, grassroots players, and broader developmental populations remain underrepresented. In addition, the lack of standardized and representative protocols for inducing and assessing MF, coupled with the predominance of subjective measures, limits comparability between studies and reduces the practical applicability of the results. These methodological inconsistencies have created a fragmented evidence base preventing practitioners from confidently implementing evidence-based monitoring systems.

Based on the recommendations identified in the studies themselves, future research should address:

- Population diversity: Future studies should include more female players, grassroots athletes, youth players, amateur players, and athletes from different competitive and developmental contexts. Although Donnan et al. (22) provides an important contribution to women's football, the evidence base remains strongly male-dominated.
- Ecologically valid MF protocols: Future research should develop standardized simulation protocols that better represent the cognitive demands of football by replicating contextual interference, incorporating decision-making under temporal pressure, and maintaining technical and tactical validity.
- Integrated subjective and objective assessment: Future studies should combine subjective tools such as VAS, RPE, and NASA-TLX with objective physiological, behavioral, and neurophysiological measures, including PVT, HRV, GPS-derived indicators, eye-tracking, EEG/ERP, pupillometry, or dual-task paradigms where feasible.
- Multidimensional performance models: Future research should adopt integrated assessment frameworks capable of evaluating physical, technical, tactical, cognitive, perceptual, and psychophysiological dimensions of performance under MF conditions.
- Longitudinal and applied monitoring designs: Future studies should track both acute and chronic MF across training microcycles, congested match schedules, recovery periods, and competitive seasons, while generating normative data for different player populations and competitive levels.

These directions could contribute to a more robust and applicable understanding of MF in football, with direct implications for performance optimization, training planning, recovery management, and prevention of performance deterioration. The development of standardized, practical assessment methods is arguably the most urgent methodological priority. Such tools would enable researchers and practitioners to generate comparable data across diverse contexts, thereby facilitating the transition from isolated experimental findings to evidence-based monitoring and management strategies for MF in applied football settings.

## 5 Conclusion

This systematic review synthesized evidence regarding the effects of MF on football performance and the methodologies currently used to induce, assess, and monitor MF in football contexts. Overall, the findings indicate that MF is associated with impairments across several dimensions of football performance, particularly cognitive processing, decision-making, technical execution, tactical behavior, and perceived exertion. However, the effects of MF on objective physical-performance outcomes appear more heterogeneous, varying according to task characteristics, sample profile, fatigue-induction protocol, and assessment method. The available literature therefore remains methodologically diverse, with substantial variability in MF induction protocols, assessment tools, and performance outcomes, limiting comparability across studies and reducing practical applicability.

Most included studies relied on controlled experimental paradigms and subjective assessment tools, whereas objective, longitudinal, and ecologically valid monitoring approaches remain comparatively underexplored. In addition, although recent evidence has expanded to include youth, amateur, collegiate, applied monitoring, and women's football contexts, the literature remains predominantly based on male and trained competitive samples. Collectively, these findings reinforce the need for more standardized, multidimensional, and ecologically valid approaches to MF assessment and monitoring in football. Advancing methodological consistency in this field may contribute to improved performance management, recovery strategies, and evidence-based decision-making in applied football settings.

## Data Availability

The data extracted and analyzed in this systematic review are available in the article and its Supplementary Material.

## References

[1] Van Cutsem J Marcora S De Pauw K Bailey S Meeusen R Roelands B. The effects of mental fatigue on physical performance: a systematic review. Sports Med. (2017) 47(8):1569–88. 10.1007/s40279-016-0672-0 28044281

[2] Sun H Soh KG Mohammadi A Wang X Bin Z Zhao Z. Effects of mental fatigue on technical performance in soccer players: a systematic review with a meta-analysis. Front Public Health. (2022) 10. 10.3389/fpubh.2022.922630 PMC9354787 35937235

[3] Sun H Soh KG Roslan S Wazir MRWN Soh KL. Does mental fatigue affect skilled performance in athletes? A systematic review. PLoS One. (2021) 16(10):e0258307. 10.1371/journal.pone.0258307 34648555 PMC8516214

[4] Smith MR Thompson C Marcora SM Skorski S Meyer T Coutts AJ. Mental fatigue and soccer: current knowledge and future directions. Sports Med. (2018) 48(7):1525–32. 10.1007/s40279-018-0908-2 29623604

[5] Kunrath CA Cardoso F Calvo TG da Costa IT. Mental fatigue in soccer: a systematic review. Rev Bras Med Esporte. (2020a) 26(2):172–8. 10.1590/1517-869220202602208206

[6] Goodman SPJ Collins B Shorter K Moreland AT Papic C Hamlin AS. Approaches to inducing mental fatigue: a systematic review and meta-analysis of (neuro)physiologic indices. Behav Res Methods. (2025) 57(4):102. 10.3758/s13428-025-02620-7 40011311 PMC11865143

[7] Pan X Soh KG Soh KL. Viable strategies for enhancing performance in ball sports by mitigating mental fatigue: a systematic review. PLoS One. (2024) 19(11):e0313105. 10.1371/journal.pone.0313105 39514612 PMC11548715

[8] Pan X Soh KG Jaafar WMW Soh KL Li M Liu H. Effects of mental fatigue on olympic ball sports performance: a systematic review. Acta Psychol (Amst). (2025) 258:105109. 10.1016/j.actpsy.2025.105109 40505574

[9] Aveiro D Martins F Pereira M Coimbra R Mendes RJ Pinto J. Mental fatigue in football: methodology and experimental protocol. Front Sports Act Living. (2026) 8:1788854. 10.3389/fspor.2026.1788854 41958822 PMC13057451

[10] Page MJ McKenzie JE Bossuyt PM Boutron I Hoffmann TC Mulrow CD. The PRISMA 2020 statement: an updated guideline for reporting systematic reviews. J Clin Epidemiol. (2021) 134:178–89. 10.1016/j.jclinepi.2021.03.001 33789819

[11] Hong QN Pluye P Fàbregues S Bartlett G Boardman F Cargo M. The mixed methods appraisal tool (MMAT) version 2018 for information professionals and researchers. Educ Inf. (2018) 34(4):285–91. 10.3233/EFI-180221

[12] Ankul SS Chandran L Anuragh S Kaliappan I Rushendran R Vellapandian C. A systematic review of the neuropathology and memory decline induced by monosodium glutamate in the Alzheimer’s disease-like animal model. Front Pharmacol. (2023) 14. 10.3389/fphar.2023.1283440 37942488 PMC10627830

[13] Field AP. Discovering Statistics Using IBM SPSS Statistics. 5th edn. Newbury Park, London: Sage (2018).

[14] Landis JR Koch GG. The measurement of observer agreement for categorical data. Biometrics. (1977) 33(1):159–74. 10.2307/2529310 843571

[15] Filipas L Borghi S La Torre A Smith MR. Effects of mental fatigue on soccer-specific performance in young players. Sci Med Footb. (2021) 5(2):150–7. 10.1080/24733938.2020.1823012 35077334

[16] Greco G Tambolini R Ambruosi P Fischetti F. Negative effects of smartphone use on physical and technical performance of young footballers. J Phys Educ Sport. (2017) 17(4):2495–501. 10.7752/jpes.2017.04280

[17] Angius L Merlini M Hopker J Bianchi M Fois F Piras F. Physical and mental fatigue reduce psychomotor vigilance in professional football players. Int J Sports Physiol Perform. (2022) 17(9):1391–8. 10.1123/ijspp.2021-0387 35477898

[18] Badin OO Smith MR Conte D Coutts AJ. Mental fatigue: impairment of technical performance in small-sided soccer games. Int J Sports Physiol Perform. (2016) 11(8):1100–5. 10.1123/ijspp.2015-0710 27003948

[19] Smith MR Fransen J Deprez D Lenoir M Coutts AJ. Impact of mental fatigue on speed and accuracy components of soccer-specific skills. Sci Med Football. (2017) 1(1):48–52. 10.1080/02640414.2016.1252850

[20] Coutinho D Gonçalves B Travassos B Wong DP Coutts AJ Sampaio JE. Mental fatigue and spatial references impair soccer players’ physical and tactical performances. Front Psychol. (2017) 8. 10.3389/fpsyg.2017.01645 28983273 PMC5613114

[21] Coutinho D Gonçalves B Wong DP Travassos B Coutts AJ Sampaio J. Exploring the effects of mental and muscular fatigue in soccer players’ performance. Hum Mov Sci. (2018) 58:287–96. 10.1016/j.humov.2018.03.004 29549745

[22] Donnan KJ Bargh MJ Swettenham L Olthof S Whitehead A. The multifaceted implications of mental fatigue on women’s football players’ performance in small-sided games. Psychol Sport Exerc. (2026) 82:103013. 10.1016/j.psychsport.2025.103013 41232737

[23] Skoki A Anic P Ljubic S Naglic A Lerga J Stajduhar I. Exploring the impact of the perceived cognitive load on the physical performance in soccer. Elektrotehniški Vestn. (2024) 91(3):108–16.

[24] Soylu Y Arslan E. Effects of mental fatigue on psychophysiological, cognitive responses, and technical skills in small-sided soccer games in amateur players. Balt J Health Phys Act. (2021) 13(7):43–50. 10.29359/BJHPA.2021.Suppl.2.05

[25] Soylu Y Ramazanoglu F Arslan E Clemente FM. Effects of mental fatigue on the psychophysiological responses, kinematic profiles, and technical performance in different small-sided soccer games. Biol Sport. (2022) 39(4):965–72. 10.5114/biolsport.2022.110746 36247954 PMC9536376

[26] Soylu Y Arslan E Akçay N Akgul MS Kilit B Lopes TR. Effects of mental fatigue on psychophysiological responses, kinematic variables and technical actions in small-sided soccer games: a time course analysis. Front Psychol. (2025) 16:1654701. 10.3389/fpsyg.2025.1654701 41112567 PMC12528091

[27] Brett A Rhodes D Grimson S Kiely J. Analysis of mental and physical fatigue over the course of a professional English premier league season in outfield players. Biol Sport. (2025) 42(3):239–45. 10.5114/biolsport.2024.133001 40656996 PMC12244382

[28] Staiano W Merlini M Romagnoli M Kirk U Ring C Marcora S. Brain endurance training improves physical, cognitive, and multitasking performance in professional football players. Int J Sports Physiol Perform. (2022) 17(12):1732–40. 10.1123/ijspp.2022-0144 36370703

[29] Smith MR Coutts AJ Merlini M Deprez D Lenoir M Marcora SM. Mental fatigue impairs soccer-specific physical and technical performance. Med Sci Sports Exercise. (2016a) 48(2):267–76. 10.1249/MSS.0000000000000762 26312616

[30] Fortes LS Lima-Junior D Nascimento-Júnior JRA Costa EC Matta MO Ferreira MEC. Effect of exposure time to smartphone apps on passing decision-making in male soccer athletes. Psychol Sport Exerc. (2019) 44:35–41. 10.1016/j.psychsport.2019.05.001

[31] Fortes LS De Lima-Junior D Fiorese L Nascimento-Júnior JRA Mortatti AL Ferreira MEC. The effect of smartphones and playing video games on decision-making in soccer players: a crossover and randomised study. J Sports Sci. (2020) 38(5):552–8. 10.1080/02640414.2020.1715181 31941416

[32] Gantois P Caputo Ferreira ME Lima-Junior D Nakamura FY Batista GR Fonseca FS. Effects of mental fatigue on passing decision-making performance in professional soccer athletes. Eur J Sport Sci. (2020) 20(4):534–43. 10.1080/17461391.2019.1656781 31424354

[33] Nishida M Okano S Ichinose A Suyama S Youn S. Daytime napping benefits passing performance and scanning activity in elite soccer players. J Sports Sci Med. (2023) 22(1):75–83. 10.52082/jssm.2023.75 36876185 PMC9982537

[34] Soltani A Memmert D Rezaie R Nazemzadegan G Jahromi M. Comparing the effect of mental fatigue-inducing models on selected cognitive and technical performance aspects in young soccer players. Sci Rep. (2026) 16:8598. 10.1038/s41598-026-39936-z 41680303 PMC12976352

[35] da Silva DC Afonso J Augusto D Petiot GH Martins Filho CC Vasconcellos F. Influence of pre-induced mental fatigue on tactical behavior and performance among young elite football players. Int J Sport Exerc Psychol. (2023) 21(5):917–29. 10.1080/1612197X.2022.2084765

[36] da Silva DC Carnevale DM Santos DAN de Novaes Andrade C Filho CCM Vasconcellos F. Mental fatigue in football: behavioral responses of players with high and low tactical performance. Retos. (2024) 51:666–71. 10.47197/retos.v51.101040

[37] Kunrath CA Nakamura FY Roca A Tessitore A Teoldo Da Costa I. How does mental fatigue affect soccer performance during small-sided games? A cognitive, tactical and physical approach. J Sports Sci. (2020b) 38(15):1818–28. 10.1080/02640414.2020.1756681 32362188

[38] Silva G Bredt S Praça G. Does experience mitigate the deleterious effect of mental fatigue on tactical performance? A study in youth soccer academies. Int J Sports Sci Coach. (2026) 21(1):154–69. 10.1177/17479541251365949

[39] Alder D Broadbent D Poolton J. The combination of physical and mental load exacerbates the negative effect of each on the capability of skilled soccer players to anticipate action. J Sports Sci. (2021) 39(9):1030–8. 10.1080/02640414.2020.1855747 33274696

[40] Smith MR Zeuwts L Lenoir M Hens N De Jong LM Coutts AJ. Mental fatigue impairs soccer-specific decision-making skill. J Sports Sci. (2016b) 34(14):1297–304. 10.1080/02640414.2016.1156241 26949830

[41] Fortes LS Barbosa BT Mortatti AL. Effect of mental fatigue on decision-making skill during simulated congested match schedule in professional soccer athletes. Curr Psychol. (2024) 43:1785–93. 10.1007/s12144-023-04437-z

[42] Rubio-Morales A Díaz-García J Berchicci M Morenas-Martín J del Campo VL García-Calvo T. The mental fatigue induced by physical, cognitive and combined effort in amateur soccer players: a comparative study using EEG. J Funct Morphol Kinesiol. (2025) 10(4):373. 10.3390/jfmk10040373 41133563 PMC12551114

