# Supplementary material for: Assessing mental fatigue in football: a systematic review
Source: Front Sports Act Living. 2026 Jun 9;8:1861469. doi: 10.3389/fspor.2026.1861469 (PMC13286965; doi:10.3389/fspor.2026.1861469)
Supplement: Supplementary file 1 [file Table1.docx]

Supplementary Material

# Supplementary Table 1. Complete database-specific search strategies used in the systematic review

| **Database** | **Date of search** | **Search string** | **Fields searched** | **Filters/limits applied** | **Records retrieved** |
| --- | --- | --- | --- | --- | --- |
| PubMed | First week of November 2024; updated on 15 May 2026 | “mental fatigue” AND (“football” OR “soccer”) AND “performance” AND (“monitor*” OR “assess*” OR “track*”) | General search field / automatic term mapping | Publication date limited to previous 12 years; abstract available; no article-type restriction applied. | N = 43 |
| Scopus | First week of November 2024; updated on 15 May 2026 | “mental fatigue” AND (“football” OR “soccer”) AND “performance” AND (“monitor*” OR “assess*” OR “track*”) | Article title, Abstract, Keywords | Publication year limited to 2014-2026, corresponding to the 12-year eligibility window at the time of the updated search. | N = 83 |
| Web of Science | First week of November 2024; updated on 15 May 2026 | “mental fatigue” AND (“football” OR “soccer”) AND “performance” AND (“monitor*” OR “assess*” OR “track*”) | Topic search (Title, Abstract, Author Keywords, Keywords Plus) | Publication years: 2014-2026; document type: Article; selected Web of Science categories/research areas related to Psychology, Physiology, Sport Sciences, Neuroscience and related fields. No database-level language filter was applied. | N = 124 |
| SportDiscus | First week of November 2024; updated on 15 May 2026 | “mental fatigue” AND (“football” OR “soccer”) AND “performance” AND (“monitor*” OR “assess*” OR “track*”) | General EBSCOhost search field | Date range: 2014-2026. No additional database-level filters were applied. | N = 129 |
| Springer Nature Link | First week of November 2024; updated on 15 May 2026 | “mental fatigue” AND (“football” OR “soccer”) AND “performance” AND (“monitor*” OR “assess*” OR “track*”) | General Search interface / broad keyword search across indexed document content; no equivalent Title/Abstract/Author Keywords or TITLE-ABS-KEY field restriction was available in the interface used. | Content type: Article; Discipline: Medicine & Public Health; Subdiscipline: Orthopedics, Rehabilitation Medicine, Human Physiology, Metabolic Diseases; publication date: 2014-2026; language: English | N = 289 |

Record counts refer to the updated search conducted on 15 May 2026 and may differ from current database outputs due to subsequent indexing updates, changes in database coverage, or search-interface modifications.

# Supplementary File 2. PRISMA 2020 Checklist

| **Section and Topic** | **Item #** | **Checklist item** | **Location where item is reported** |
| --- | --- | --- | --- |
| **TITLE** | | |  |
| Title | 1 | Identify the report as a systematic review. | Title page |
| **ABSTRACT** | | |  |
| Abstract | 2 | See the PRISMA 2020 for Abstracts checklist. | Abstract |
| **INTRODUCTION** | | |  |
| Rationale | 3 | Describe the rationale for the review in the context of existing knowledge. | Introduction |
| Objectives | 4 | Provide an explicit statement of the objective(s) or question(s) the review addresses. | Introduction, final paragraph |
| **METHODS** | | |  |
| Eligibility criteria | 5 | Specify the inclusion and exclusion criteria for the review and how studies were grouped for the syntheses. | Methods, Section 2.2 Eligibility Criteria |
| Information sources | 6 | Specify all databases, registers, websites, organisations, reference lists and other sources searched or consulted to identify studies. Specify the date when each source was last searched or consulted. | Methods, Section 2.1 Search Strategy and Information Sources; Supplementary Table 1 |
| Search strategy | 7 | Present the full search strategies for all databases, registers and websites, including any filters and limits used. | Methods, Section 2.1 Search Strategy and Information Sources; Supplementary Table 1 |
| Selection process | 8 | Specify the methods used to decide whether a study met the inclusion criteria of the review, including how many reviewers screened each record and each report retrieved, whether they worked independently, and if applicable, details of automation tools used in the process. | Methods, Section 2.2 Eligibility Criteria; Figure 1 |
| Data collection process | 9 | Specify the methods used to collect data from reports, including how many reviewers collected data from each report, whether they worked independently, any processes for obtaining or confirming data from study investigators, and if applicable, details of automation tools used in the process. | Methods, Section 2.4 Reviewing Studies and Collecting Data; Section 2.5 Analysing Studies and Extracting Data |
| Data items | 10a | List and define all outcomes for which data were sought. Specify whether all results that were compatible with each outcome domain in each study were sought (e.g. for all measures, time points, analyses), and if not, the methods used to decide which results to collect. | Methods, Section 2.4-2.5; Results, Sections 3.1-3.3; Table 2 |
|  | 10b | List and define all other variables for which data were sought (e.g. participant and intervention characteristics, funding sources). Describe any assumptions made about any missing or unclear information. | Methods, Section 2.4-2.5; Table 2 |
| Study risk of bias assessment | 11 | Specify the methods used to assess risk of bias in the included studies, including details of the tool(s) used, how many reviewers assessed each study and whether they worked independently, and if applicable, details of automation tools used in the process. | Methods, Section 2.3 Quality Assessment; Table 1 |
| Effect measures | 12 | Specify for each outcome the effect measure(s) (e.g. risk ratio, mean difference) used in the synthesis or presentation of results. | Not applicable; no meta-analysis was conducted, and no pooled effect measures were calculated. |
| Synthesis methods | 13a | Describe the processes used to decide which studies were eligible for each synthesis (e.g. tabulating the study intervention characteristics and comparing against the planned groups for each synthesis (item #5)). | Methods, Section 2.5 Analysing Studies and Extracting Data |
|  | 13b | Describe any methods required to prepare the data for presentation or synthesis, such as handling of missing summary statistics, or data conversions. | Not applicable; no quantitative data preparation, conversion, or meta-analysis was conducted. |
|  | 13c | Describe any methods used to tabulate or visually display results of individual studies and syntheses. | Methods, Section 2.5 Analysing Studies and Extracting Data |
|  | 13d | Describe any methods used to synthesize results and provide a rationale for the choice(s). If meta-analysis was performed, describe the model(s), method(s) to identify the presence and extent of statistical heterogeneity, and software package(s) used. | Methods, Section 2.5 Analysing Studies and Extracting Data |
|  | 13e | Describe any methods used to explore possible causes of heterogeneity among study results (e.g. subgroup analysis, meta-regression). | Not applicable; no meta-analysis, subgroup analysis, or meta-regression was conducted. |
|  | 13f | Describe any sensitivity analyses conducted to assess robustness of the synthesized results. | Not applicable; no sensitivity analysis was conducted. |
| Reporting bias assessment | 14 | Describe any methods used to assess risk of bias due to missing results in a synthesis (arising from reporting biases). | Not assessed; no formal reporting bias assessment was conducted. |
| Certainty assessment | 15 | Describe any methods used to assess certainty (or confidence) in the body of evidence for an outcome. | Not assessed; certainty of evidence was not formally assessed. |
| **RESULTS** | | |  |
| Study selection | 16a | Describe the results of the search and selection process, from the number of records identified in the search to the number of studies included in the review, ideally using a flow diagram. | Results, Section 3; Figure 1 |
|  | 16b | Cite studies that might appear to meet the inclusion criteria, but which were excluded, and explain why they were excluded. | Results, Section 3; Figure 1 |
| Study characteristics | 17 | Cite each included study and present its characteristics. | Results, Table 2 |
| Risk of bias in studies | 18 | Present assessments of risk of bias for each included study. | Results, methodological quality assessment section/table |
| Results of individual studies | 19 | For all outcomes, present, for each study: (a) summary statistics for each group (where appropriate) and (b) an effect estimates and its precision (e.g. confidence/credible interval), ideally using structured tables or plots. | Results, Sections 3.1-3.3; Table 2 |
| Results of syntheses | 20a | For each synthesis, briefly summarise the characteristics and risk of bias among contributing studies. | Results, Sections 3.1-3.3; Discussion, Section4 |
|  | 20b | Present results of all statistical syntheses conducted. If meta-analysis was done, present for each the summary estimate and its precision (e.g. confidence/credible interval) and measures of statistical heterogeneity. If comparing groups, describe the direction of the effect. | Not applicable; no meta-analysis or statistical synthesis was conducted. |
|  | 20c | Present results of all investigations of possible causes of heterogeneity among study results. | Not applicable; no formal heterogeneity investigation was conducted. |
|  | 20d | Present results of all sensitivity analyses conducted to assess the robustness of the synthesized results. | Not applicable; no sensitivity analysis was conducted. |
| Reporting biases | 21 | Present assessments of risk of bias due to missing results (arising from reporting biases) for each synthesis assessed. | Not assessed; no formal reporting bias assessment was conducted. |
| Certainty of evidence | 22 | Present assessments of certainty (or confidence) in the body of evidence for each outcome assessed. | Not assessed; certainty of evidence was not formally assessed. |
| **DISCUSSION** | | |  |
| Discussion | 23a | Provide a general interpretation of the results in the context of other evidence. | Discussion, Section 4 |
|  | 23b | Discuss any limitations of the evidence included in the review. | Discussion, Section 4; Section 4.1 Gaps in the Literature and Future Recommendations |
|  | 23c | Discuss any limitations of the review processes used. | Discussion, Section 4; Section 4.1 Gaps in the Literature and Future Recommendations |
|  | 23d | Discuss implications of the results for practice, policy, and future research. | Discussion, Section 4; Section 4.1 Gaps in the Literature and Future Recommendations; Conclusion |
| **OTHER INFORMATION** | | |  |
| Registration and protocol | 24a | Provide registration information for the review, including register name and registration number, or state that the review was not registered. | Methods, Section 2.1 Search strategy |
|  | 24b | Indicate where the review protocol can be accessed, or state that a protocol was not prepared. | Methods, Section 2.1 Search strategy |
|  | 24c | Describe and explain any amendments to information provided at registration or in the protocol. | Not applicable; no registered protocol was available. |
| Support | 25 | Describe sources of financial or non-financial support for the review, and the role of the funders or sponsors in the review. | Funding statement |
| Competing interests | 26 | Declare any competing interests of review authors. | Conflict of interest statement |
| Availability of data, code and other materials | 27 | Report which of the following are publicly available and where they can be found: template data collection forms; data extracted from included studies; data used for all analyses; analytic code; any other materials used in the review. | Data availability statement; Supplementary Materials |

*From:* Page MJ, McKenzie JE, Bossuyt PM, Boutron I, Hoffmann TC, Mulrow CD, et al. The PRISMA 2020 statement: an updated guideline for reporting systematic reviews. BMJ 2021;372:n71. doi: 10.1136/bmj.n71
